# A Comprehensive Experimental and Theoretical Study on the [{(η^5^-C_5_H_5_)_2_Zr[P(µ-PNEt_2_)_2_P(NEt_2_)_2_P]}]_2_O Crystalline System

**DOI:** 10.3390/molecules26237282

**Published:** 2021-11-30

**Authors:** Agnieszka Łapczuk-Krygier, Katarzyna Kazimierczuk, Jerzy Pikies, Mar Ríos-Gutiérrez

**Affiliations:** 1 Institute of Organic Chemistry and Technology, Krakow University of Technology, Warszawska St. 24, 31-155 Krakow, Poland; 2 Department of Inorganic Chemistry, Faculty of Chemical, Gdansk University of Technology, G. Narutowicza St. 11/12, 80-233 Gdansk, Poland; katarzyna.kazimierczuk@pg.edu.pl (K.K.); jerpikie@pg.edu.pl (J.P.); 3 Department of Organic Chemistry, University of Valencia, Dr. Moliner 50, 46100 Valencia, Spain

**Keywords:** X-ray crystal structure, zirconocene, phosphetane, ELF, QTAIM

## Abstract

The structure of tetraphosphetane zirconium complex C_52_H_100_N_8_OP_10_Zr_2_ **1** was determined by single crystal X-ray diffraction analysis. The crystal belongs to the monoclinic system, space group P21/c, with a = 19.6452(14), b = 17.8701(12), c = 20.7963(14)Å, α = γ = 90°, β = 112.953(7)°, V = 6722.7(8)Å3, Z = 4. The electronic structure of the organometallic complex has been characterized within the framework of Quantum Chemical Topology. The topology of the Electron Localization Function (ELF) and the electron density according to the Quantum Theory of Atoms in Molecules (QTAIM) show no covalent bonds involving the Zr atom, but rather dative, coordinate interactions between the metal and the ligands. This is the first reported case of a Zr complex stabilized by an oxide anion, anionic cyclopentadienyl ligands and rare tetraphosphetane anions.

## 1. Introduction

This work is a continuation of our comprehensive study of phosphorous complexes. The chemistry of the metalocenes with phourous ligands is very interesting, but, unfortunately, their chemical properties are still rather poorly known. The metallocenes bearing one or two cyclopentadienyl ligands are an important class of the new generation of polymerization catalysts for ethylene polymerization [1,2,3,4], asymmetric imine reduction [5], atom transfer radical polymerization [6,7], the dehydrocoupling of dimethylamineborane [8] and imine hydrogenation [9].

The structure and bonding properties of metallocenes and phosphoroorganic molecules have recently received much attention. Although a number of zirconocene complexes with phosphorous ligands have been reported [10], there have been no reports on the structural characterization of compounds with oxygen links, despite those of the polyphosphorus derivatives being known.

Reactions of lithium derivatives of diphosphanes R_2_PP(SiMe_3_)Li with zirconocenes lead to the formation of phosphanylophosphinidene (R_2_P-P) and phosphanylphosphido (R_2_P-(R’)P) complexes. It has been shown that thermal decomposition of phosphanylphosphido complexes yields polyphosphorus compounds [11] and if R = Et_2_N or iPr_2_N, it leads to different phosphetanes [12]. The reactivity of alkali metal phosphides towards zirconocenes has been the subject of thorough studies [11,12,13,14]. 

**Figure 1:**
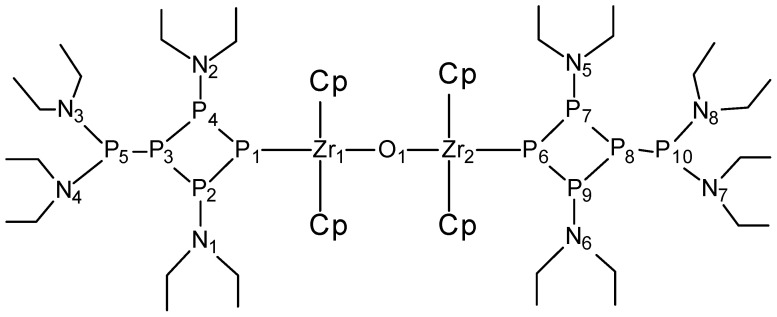
Structure of [{(η^5^-C_5_H_5_)_2_Zr[P(µ-PNEt_2_)_2_P(NEt_2_)_2_P]}]_2_O (**1**).

Here, we report on interesting an dimeric complex, [{(η^5^-C_5_H_5_)_2_Zr[P(µ-PNEt_2_)_2_P(NEt_2_)_2_P]}]_2_O (**1**), with two phosphetane moieties and two zirconocene groups (Figure 1). Formation of polyphosphorus complexes was earlier observed [11,14], but complexes of phosphetanes as ligands in transition metals complexes are very rare.

## 2. Results and Discussion

### 2.1. Synthesis and X-ray Study 

**Scheme 1:**
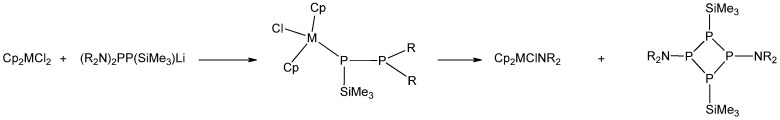
Reactions of lithium derivatives of diphosphanes (Et_2_N)_2_PP(SiMe_3_)Li with metalocene dichloride (M = Hf, R = Et_2_N), yielding a phosphanylphosphido ((Et_2_N)_2_P-(Me_3_Si)P) complex, whose thermal decomposition leads to tetraphosphetane.

Our previous research [12,15] has focused on reactions of [Cp_2_ZrCl_2_] with lithium derivatives of diphosphanes R_2_PP(SiMe_3_)Li. We have confirmed the substitution of one or two chlorido ligands and formation of phosphanylphosphido (R_2_P-(Me_3_Si)P) or phosphanylophosphinidene (R_2_P-P) complexes with a zirconocene group. For R = Et_2_N or ^i^Pr_2_N, the phosphanylphosphido complexes are not always stable, and in reaction solutions, different phosphetanes can be found. Scheme 1 [12] shows the similar reaction of [Cp_2_HfCl_2_] with (Et_2_N)_2_P-P(SiMe_3_)Li, yielding almost solely the related phosphetane Et_2_NP(PSiMe_3_)_2_PNEt_2_.

Reactions of CpZrCl_3_ with (R_2_N)_2_P-P(SiMe3)Li (R = Et or ^i^Pr) yield a variety of phosphetanes: R_2_NP(PSiMe_3_)_2_PNR_2_, R_2_NP-P(PSiMe_3_)_2_P-PNR_2_ and R_2_NP-P(PNR_2_)_2_PSiMe_3_ [12]. It should be stressed that the formation of R_2_NP(PSiMe_3_)_2_PNR_2_ can be easy rationalized in terms of Scheme 1 and very likely involves an intermediate phosphanylphosphido complex. The formations of R_2_NP-P(PSiMe_3_)_2_P-PNR_2_ and R_2_NP-P(PNR_2_)_2_PSiMe_3_ are more complicated. Recently, unexpected ^i^Pr_2_NP-P(PN^i^Pr_2_)_2_PSiMe_3_ was formed in high yield in a reaction of [NacNacFe(µ-Cl)_2_Li(DME)_2_] with (^i^Pr_2_N)_2_PP(SiMe_3_)Li. The side products, P(SiMe_3_)_3_ and (^i^Pr_2_N)_2_PP(N^i^Pr_2_)_2_, pointed to the oxidation of (^i^Pr_2_N)_2_PP(SiMe_3_)Li involving the TM complex as a driving force of this reaction [15]. 

Dimeric complex **1** incorporated two ZrCp_2_ moieties in one molecule, although CpCp *ZrCl_2_ was used as a starting complex. There was likely an initial rearrangement in the reaction mixture prior to the phosphetane (Et_2_N)_2_PP(PNEt_2_)_2_PSiMe_3_ formation. In our studies, we often isolate Cp_2_ZrCl_2_ from the reaction mixture while only CpCp*ZrCl_2_ is used in the reaction (1)

2 Cp*CpZrCl_2_→Cp_2_ZrCl_2_ + Cp*_2_ZrCl_2_ (1)

**Scheme 2:**
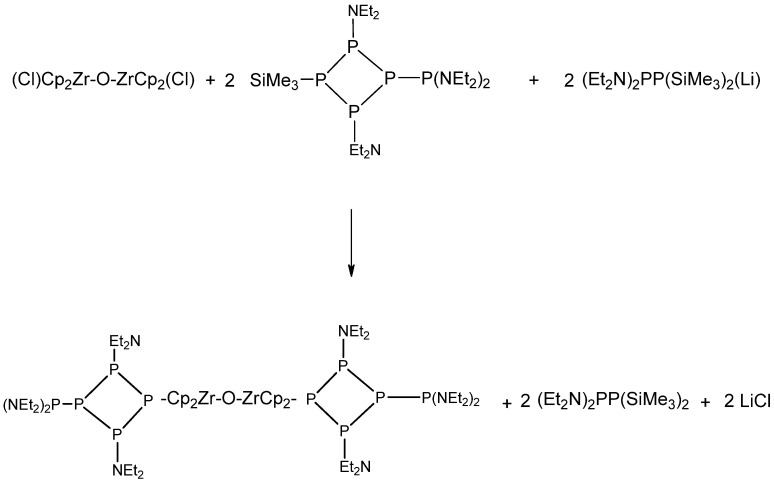
Reaction of (Cl)Cp_2_Zr-*O*-ZrCp_2_(Cl) with the phosphetane and an excess of (Et_2_N)_2_PPSiMe_3_(Li) yielding complex **1**, LiCl and (Et_2_N)_2_PP(SiMe_3_)_2_.

The next step is the reaction of (Cl)Cp_2_Zr-O-ZrCp_2_(Cl) with the phosphetane and an excess of (Et_2_N)_2_PPSiMe_3_(Li) yielding complex **1**, LiCl and (Et_2_N)_2_PP(SiMe_3_)_2_ (Scheme 2).

Experiments with Cp*CpZrCl_2_ have shown that it is far less reactive than Cp_2_ZrCl_2_ towards lithium salts of diphosphanes and it likely did not react.

The Zr-O-Zr moiety more likely originates from the decomposition of THF by zirconium compounds or from hydrolysis with traces of water of zirconocene dichloride. Similar compounds were obtained by subjecting a solution of zirconocenederivatives in a toluene–hexane mixture to slow hydrolysis in air [16,17], or they can be prepared from µ-oxo dichloride complexes [{Cp_2_Zr(Cl)}_2_O] [18]. These compounds are very susceptible to hydrolysis and the formation of coordination polymers.

The study of a reaction solution with ^31^P-NMR displayed a complicated mixture of partially not identified species and indicated that Et_2_NP(PSiMe_3_)_2_PNEt_2_ was not formed; thus, the reaction similar to Scheme 1 did not proceed. 

**Figure 2:**
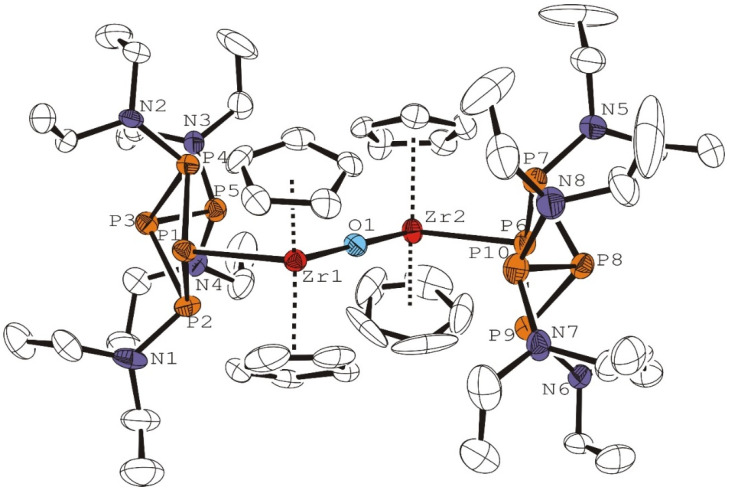
ORTEP3 drawing of the title compound (ellipsoids for non-H atoms are drawn at the 50% probability level).

**Figure 3:**
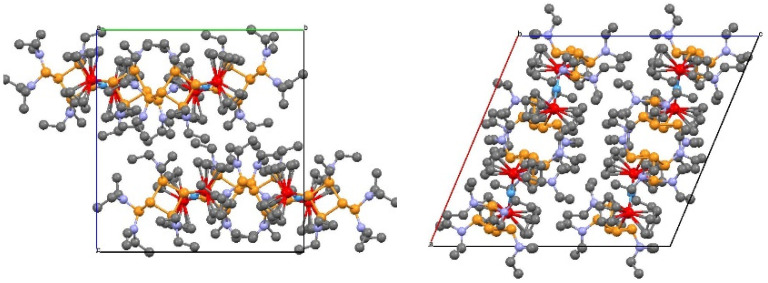
The crystal packing of the title complex, viewed along a and b axes.

The molecular structure of **1** shown in Figure 2 consists of two nearly identical (η^5^-C_5_H_5_)_2_Zr molecular units linked together by an oxygen. The oxygen occupies one of four metal coordination sites, and with the polyphosphorus group and the two centroids of the cyclopentadienyl rings, completes a distorted tetrahedral geometry around each zirconium center (Figure 3).

The Cp_centroid_-Zr-Cp_centroid_ angle of 131.0° is normal for zirconocene complexes, and the average Zr-Cp_centroid_ distance of 2.21 Å is comparable with those of other derivatives. The angle between Zr-O-Zr depends on the R-group (Table 1 and Table 2).

The phosphorous skeleton indicates a butterfly structure similar to 2,4-bis[bis(diisopropylamino)phosphanyl]-1,2,3,4-tetraphosphabicyclo- [1.1.0]butane [14]. The distances between the centers of cyclopentadienyl rings and zirconium atoms are 2.235 Å and 2.247 Å for (Zr1) and 1.251 Å, 2.251 Å and 2.245 Å for (Zr2). The angles between Cp-centroids and atoms are 129.395° in Cp_2_Zr1 and 128.10° in Cp_2_Zr_2_ (Table 3 and Table 4).

### 2.2. Theoretical Study 

Although the nuclei position and cristallographic data of complex **1** have been clearly characterized by X-ray crystallography, the nature of the chemical bonding in its framweork remains unknown. Thus, in order to gain some insight into the electronic structure and bonding pattern of complex **1**, the geometry of a reduced computational model **1′** in which the ethyl groups at **1** were substituted by hydrogen atoms was first optmized and compared to both experimetal and theoretical data of **1**. Then, the bonding pattern of model complex **1′** was characterized within the Quatum Chemical Topology [20] perspective, through the topological analysis of the electron localization function (ELF) [21] and the electron density, by means of the Quantum Theory of Atoms in Molecules (QTAIM) [22,23,24].

### 2.3. Geometrical Analysis of Complex **1** and Reduced Model **1′**

**Figure 4:**
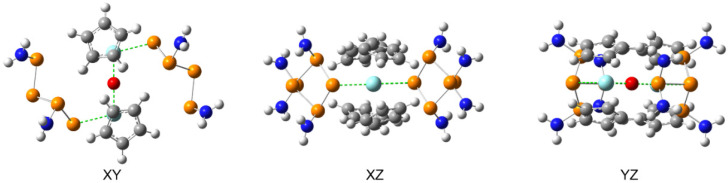
Views of the *ω*B97X-D/Def2TZV gas phase-optimized geometry of model **1′** along planes XY, XZ and YZ.

In order to check the suitability of reduced model **1′** to represent the bonding pattern of complex **1**, the X-ray and *ω*B97X-D/Def2TZV geometries of the main motif of **1** and the *ω*B97X-D/Def2TZV-optimized geometry of reduced model **1′** were compared. Table 5 gathers the most relevant data, namely the distances between each Zr atom and the surrounding tetraphosphetane, cyclopentadienyl and oxygen systems, while Figure 4 displays the optimized geometry of reduced model **1′**. 

As can be observed from Table 5, the most remarkable difference between the experimental and the computed geometries is that the distances are slightly shorter in the former as a consequence of the crystal packing. The DFT-optimized geometry of **1** is very similar to the experimental one; the Zr-X (with X = Cp, P or O) distances differ by 0.06 Å at most and three of the four O-Zr-Cp angles vary by less than 2°. On the other hand, the geometry of the reduced model **1′** is almost equivalent to the DFT geometry of **1**; the most relevant distances change by less than 0.02 Å. When compared to the experimental data, the geometry of the reduced model **1′** fits, as well as the DFT geometry of **1**, and even improves some of the angles. Consequently, these data show that the reduced model **1′** can be used as a representative case study to illustrate the bonding pattern of **1**. 

#### 2.3.1. ELF Topological Analysis of **1′**

**Figure 5:**
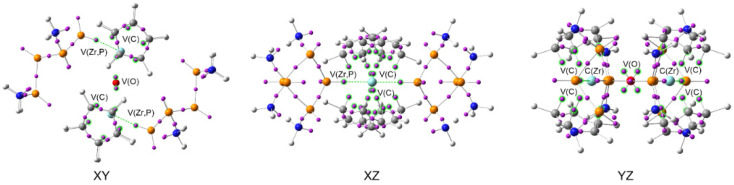
ELF attractor positions of the core and valence basins of **1′** viewed along planes XY, XZ and YZ. Relevant attractors are highlighted in green.

**Figure 6:**
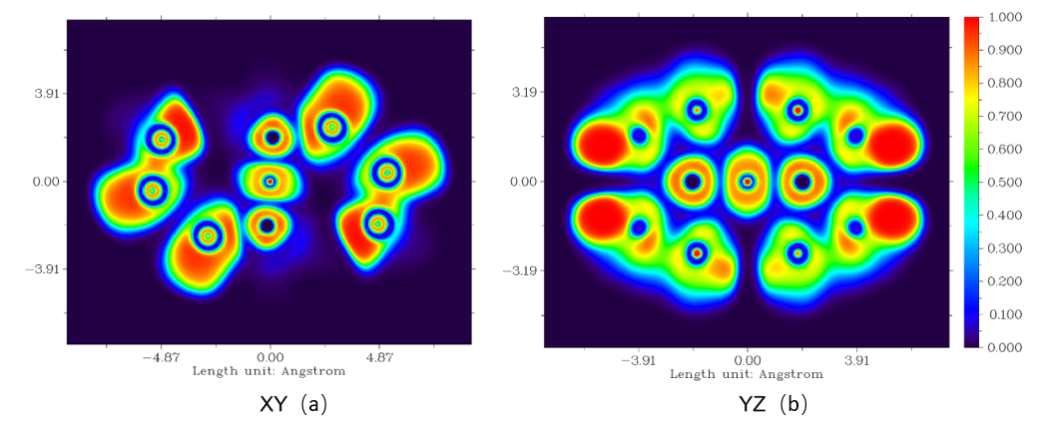
Color-filled maps of the ELF of **1′** along planes XY (**a**) and YZ (**b**).

The quantum chemical analysis of Becke and Edgecombe’s ELF [25] is an appealing procedure that provides a straightforward connection between the electron density distribution and the chemical structure [26,27]. Thus, in order to shed light onto the bonding characteristics of complex **1**, the ELF of **1′** was topologically analyzed. The ELF attractor positions of the core and valence basins of **1′** are shown in Figure 5, the most relevant valence basin populations are given in Table 6 and 2D color-filled maps of the ELF are represented in Figure 6. 

Within the ELF context, monosynaptic basins, labelled V(A), are associated with non-bonding regions, while disynaptic basins, labelled V(A,B), connect the core of two nuclei A and B and thus correspond to a bonding region between A and B [28]. The topological analysis of the ELF of **1′** shows the presence of four V(O) monosynaptic basins integrating a total population of 7.48 e, two C(Zr) core basins integrating 37.34 e, five V(C) monosynaptic basins integrating an average total population of 1.21 e at each Cp ring and two V(Zr,P) disynaptic basins integrating 1.95 e (see Table 6). Therefore, the ELF basin synapticities and populations suggest the presence of an O^2−^ oxide anion, four Cp^1−^ anions and two Zr^2+^ cations, which seem to be covalently bound only to the closer P phosphorus atom of the contiguous tetraphosphetane systems. 

In order to further ascertain the ELF bonding pattern of **1′**, the 2D color-filled maps of the ELF along planes XY and YZ are represented in Figure 6. The 2D color-filled map along plane XY emphasizes the P-Zr1-O-Zr2-P system (Figure 6a), while the one along plane YZ highlights the Cp_2_-Zr1-O-Zr2-Cp_2_framework (Figure 6b). Both Figure 6a,b show that the aformentioned sets of nuclei are separated from each other by light blue color regions associated with very low ELF values of 0.1–0.2, which means that there are no covalent interactions between them. Interestingly, Figure 6b also evidences the polarized structure of the Cp anions towards the Zr cations and the different shape of the ELF domains depending on the nuclei nature. In this sense, the deformation of the ELF domains of the O and P nuclei around the Zr nucleus is noteworthy.

These pictures suggest that there is no covalent Zr-P bond, despite the disynaptic assignation of the V(Zr,P) basin between the P and Zr nuclei, thus emphasizing the need for a deeper analysis.

#### 2.3.2. QTAIM Analysis of **1′**

In order to complement the topological analysis of the ELF and clarify the bonding pattern of **1′**, the topology of the electron density distribution was analyzed within the QTAIM [23,24]. The evaluation of the sign of the Laplacian of the electron density in combination with other indicators at the bonding critical points (BCPs), such as the electron density (*ρ*_cp_), the Lagrangian kinetic energy (G_cp_) and the local energy density (H_cp_), can offer a valuable insight into the the types of bonds that they are signatures of. Table 7 provides a summary of the topological indicators and features that characterize the atomic interactions [25].

The atomic interactions can be classified [26,27,28] as open-shell (shared) interactions (∇^2^*ρ*_cp_ < 0, H_cp_ ≪ 0), transit (intermediate) interactions (∇^2^*ρ*_cp_ > 0, H_cp_ < 0) and closed-shell interactions (∇^2^*ρ*_cp_ > 0, H_cp_ > 0). Open-shell interactions involve covalent and polar covalent bonds [27,29]; intermediate interactions are partially covalent interactions which include coordinate (dative) bonds, strong hydrogen bonds, metallic bonds, etc. [26,29,30]; and closed-shell interactions include ionic bonds and weak intermolecular interactions, such as weak and medium hydrogen bonds, van der Waals interactions, etc. [26,29].

Additionally, atomic interactions can be classified by the bond degree parameter BD = H_cp_/*ρ*_cp_ (the total energy per electron at BCP) [27,28]. For non-covalent closed-shell interactions, the BD parameter is positive and indicates a softening degree (SD): the weaker and more closed-shell in nature the interactionis, the greater the SD magnitude. For shared and intermediate (partially covalent) interactions, the BD parameter is negative and indicates the covalence degree (CD) of the interactions, i.e., the stronger and more covalent the interactions are, the greater the CD magnitude.

The value of electron density at BCP is also an important characteristic [23,31]. Covalent interactions exhibit *ρ*_cp_ > 0.14 a.u. [26], partially covalent (intermediate) interactions are characterized by 0.04 < *ρ*_cp_ < 0.12 a.u. [26,32] and closed-shell (electrostatic) interactions are characterized by *ρ*_cp_ < 0.04 a.u. [33]. 

The balance between the kinetic electron energy density G_cp_ and the potential electron energy density V_cp_ has been also used to reveal the nature of interactions. If |V_cp_|/G_cp_> 2, then the interaction is covalent in nature; if 1 < |V_cp_|/G_cp_< 2, then the interaction is only partially covalent; and if |V_cp_|/G_cp_< 1, then the nature of the interaction is purely non-covalent [30].

**Figure 7:**
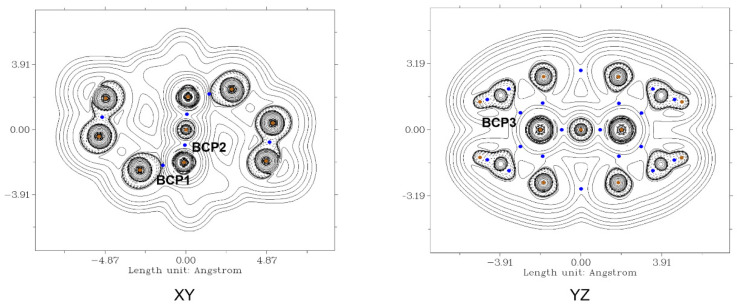
Contour-line maps of the Laplacian of the electron density ∇^2^ρ_cp_ of **1′** on the molecular planes XY and YZ.

The QTAIM topological indicators at the BCP associated with the Zr-P (BCP1), Zr-O (BCP2) and Zr-Cp (BCP3) regions of **1′** are gathered in Table 8, while Figure 7 shows the contour-line maps of the Laplacian of the electron density ∇^2^*ρ*_cp_ of **1′** on the molecular planes XY and YZ. Note that only these regions have been considered as there is no ambiguity about the pure covalent character of the Cp C-C and C-H bonds and the tetraphosphetane P-P, P-N and N-H bonds. The three BCPs present ∇^2^*ρ*_cp_ > 0, H_cp_ < 0 and 0.04 < *ρ*_cp_ < 0.12 a.u., indicating that the interactions involving the Zr atom are intermediate interactions between typical covalent and ionic bonds. In addition, Table 7 provides a way to distinguish between different types of intermediate interactions. Thus, the ∇^2^*ρ*_cp_ > 0, the G_cp_/*ρ*_cp_ ~ 1 and the H_cp_ ~ 0, together with the discard of any metallic bond, suggest that the considered interactions correspond to dative or coordinate bonds. According to the BD parameter, both Zr-P (BD = −0.26) and Zr-O (BD = −0.22) dative bonds have more covalent character than the Zr-Cp coordinate bond (BD = −0.10). Indeed, the |V_cp_|/G_cp_ quantity suggests that while both Zr-P and Zr-O interactions fit well as partial covalent interactions, the Zr-Cp one is on the borderline between intermediate and ionic interactions. 

On the other hand, delocalization indices (DI) provide a measure of the Fermi correlation shared between atomic basins and hence of the number of electrons shared. The DI values for complex **1′** are given in Table 8. As can be observed, the DI values increase in the order Zr-P < Zr-O < Zr-Cp, indicating a higher shared density between the Zr and Cp. This tendency contrasts with the inverse covalency trend characterized by the BD and |V_cp_|/G_cp_ indicators, which suggest an almost ionic Zr-Cp interaction, but can be explained by the fact that the sum of DI between Zr and the five C and H atoms of Cp is considered; the DI at one single BCP between Zr and one of the Cp carbon atoms is 0.208. The higher DI in Zr-O than in Zr-P, despite its lower BD, can also be explained by the higher electronegativity of the oxide anion, which might polarize the density of the Zr cation more into the former region. 

The source function (SF) provides a measure of the relative importance of each atomic basin’s contribution to the density at a reference point, which are usually the CPs. Gatti et al. [34] proposed that the integrated form of the SF provides pertinent chemical information. The percentages of the integrated SF are given in Table 9. These values show that nearly the 66% of the density at the three Zr-O, Zr-P and Zr-Cp interactions is recovered from the Zr involved in such interactions, while about 18% comes from the other Zr. The O, P and Cp basins contribute by about 10% in their respective interactions. This finding contrasts with the traditional conception that in dative or coordinate bonds, most of the partially shared electron density comes from the donor atom.

Finally, the Bader charges were computed and analyzed. The Bader charges are +2.17 (Zr), −1.26 (O), −0.47 (P) and −0.49 (Cp) e. These results suggest the presence of two Zr^2+^ cations, each of them stabilized by one P^1/2−^ phosphorous anion, two Cp^1/2−^ cyclopentadienyl anions and one O^1−^ oxide anion in common. 

This QTAIM picture of the bonding pattern of **1′** is not only in complete agreement with the topological analysis of the ELF—which gives no covalent Zr-O and Zr-Cp bonds but only monosynaptic basins associated with the oxygen and Cp rings—but also corrects the misleading disynaptic assignation of the basin found between Zr and P and allows adding valuable, complementary information about the nature of bonding in the regions of interest. Note that in spite of the analogy of dative bonds with covalent bonds, the former are distinguished by their significant polarity, lesser strength and greater length, in such a way that the electron density mostly belongs to one of the two atoms involved. It is worth emphasizing, however, that while the ELF monosynaptic basins belong to the O, P and C(Cp) atoms, the percentage contributions to the integrated SF indicate that the electron density in the regions between those atoms and Zr comes mainly from the metal. 

## 3. Materials and Methods

### 3.1. Synthesis

A standard Schlenk technique and an inert atmosphere (argon) were employed for the synthesis and subsequent manipulations. Toluene and THF were dried over Na/benzophenone and distilled under nitrogen. A solution of (Et_2_N)_2_PPSiMe_3_Li [35] (1 mmol) in THF was added to CpCp*ZrCl_2_ [36] (0.5 mmol) in THF. The mixture turned initially dark red and rapidly discolored. Solvent was evaporated under vacuum, dry residue was dissolved in pentane and LiCl was filtered. This pentane solution was concentrated, and after several days at −70 °C, a small amount of red crystals of compound **1** was deposited. When the reaction mixture was heated (50 °C, 4 h) the yield of isolated compound **1** increased (yield: 5–10%). The attempts to study compound **1** with NMR in solution did not succeed because of low solubility of this compound. Compound **1** is extremely sensitive and easily hydrolyzes on air. Elemental analysis and IR spectra are included in the Supplementary Materials.

### 3.2. X-ray Crystallography

Experimental diffraction data were collected on a KM4CCD kappa-geometry diffractometer, equipped with a Sapphire2 CCD detector. An enhanced X-ray MoKα radiation source with a graphite monochromator was used. Determination of the unit cells and data collection were carried out at 298 K. Data reduction, absorption correction, space group determination, solution and refinement were performed using the CRYSALISPRO software package [37]. The structures were solved by direct methods and refined by full-matrix least-squares on F^2^ (all data) using the SHELXL program package [38]. 

Crystallographic data for the structure reported here have been deposited with the Cambridge Crystallographic Data Centre (deposition no. CCDC-1059036). These data can be obtained free of charge via http://www.ccdc.cam.ac.uk/perl/catreq.cgi (accessed on 4 October 2021) (or from the CCDC, 12 Union Road, Cambridge CB2 1EZ, U.K.; Fax: (+44) 1223-336-033; e-mail: deposit@ccdc.cam.ac.uk).

### 3.3. Computational 

DFT calculations were performed with the Gaussian 16 suite of programs [39], using the hybrid ωB97X-D functional [40] together with the Def2TZV triple zeta valence basis set [41] and a singlet electronic configuration. The Berny method was used in optimizations [42,43].

The topology of the ELF [21] of the ωB97X-D/Def2TZV monodeterminantal wavefunctions was carried out using the TopMod [44] package with a cubical grid of step size of 0.1 Bohr, while QTAIM [23,24] analysis and ELF color-filled maps were performed using Multiwfn software [45]. The GaussView program [46] was used to visualize molecular geometries and ELF attractor positions. 

## 4. Conclusions

In summary, we obtained and determined the X-ray structure of [{(η^5^-C_5_H_5_)_2_Zr[P(µ-PNEt_2_)_2_P(NEt_2_)_2_P]}]_2_O. This compound was obtained in a reaction of Cp*CpZrCl_2_ with (Et_2_N)_2_PPSiMe_3_(Li) in THF. We want to stress that complexes which bound phosphetane groups via phosphorus–transition metal bonds are extremely rare. 

The theoretical calculations are in agreement with experimental data and show that the interactions involving the Zr metal in the main motif of crystal **1** can be characterized as dative, coordinate bonds in which the non-bonding electron density mainly belongs to the donor (O^1−^, P^1/2−^ or Cp^1/2−^) anions. Interestingly, the partially shared electron density in the Zr-O, Zr-P and Zr-Cp coordinate bonds mainly comes from the acceptor Zr^2+^ metal cations, increasing the covalent character in the order Zr-Cp < Zr-O < Zr-P. This bonding pattern revealed by QCT methodology suggests that the electronic structure of this type of metal complexes differs from the usual conception based on covalent M-X bonds.

## Figures and Tables

**Table 1:** Bond angles between Zr-O-Zr and Zr-O distance in [(η^5^-C_5_H_5_)_2_Zr]_2_O.

Compound	M-O (Å)	O-M-O (°)	Ref.
{(η^5^-C_5_H_5_)_2_Zr[P(µ-PNEt_2_)_2_P(NEt_2_)_2_P]}_2_O	1.960(4) 1.955(4)	179.3(3)	This work
[(η^5^-C_5_H_5_)_2_Zr(SC_6_H_5_)]_2_O	1.968(3) 1.964(3)	165.8(2)	[16]
[(η^5^-C_5_H_5_)_2_Zr(NCO)]_2_O	1.949(3) 1.942(3)	165.7(2)	[19]
[(η^5^-C_5_H_5_)_2_Zr(OC(OtBu)=CMe_2_)]_2_O	1.966(1) 1.972(1)	174.9(1)	[18]

**Table 2:** Crystallographic characteristics and the X-ray data collection and structure-refinement parameters for C_52_H_100_N_8_OP_10_Zr_2_.

Empirical Formula	C_52_H_100_N_8_OP_10_Zr_2_
Formula weight	1345.54
Temperature/K	120(2)
Wavelength/Å	0.71073 (Mo K_α_)
Crystal system	monoclinic
Space group	*P* 2_1_/*n*
*a*/Å	19.6452(14)
*b*/Å	17.8701(12)
*c*/Å	20.7963(14)
*α*/deg	90
*β*/deg	112.953(7)
γ/deg	90
*V*/Å ^3^	6722.7(8)
*Z*	4
*D*_c_/Mg m^–3^	1.329
*μ*/mm^–1^	0.588
*F*(000)	2824
Crystalsize/mm	0.0673 × 0.0423 × 0.0141
*Θ*range/deg	2.25 to 25.5
Index ranges	−21 ≤ h ≤ 23 −21 ≤ k ≤ 21 −25 ≤ l ≤ 23
Reflections collected/unique	47963/12510[*R*(int) = 0.1454]
Data/restraints/parameters	12510/0/658
Goodness of fit on *F*^2^	0.805
Final *R* indices [*I*>2σ(*I*)]	*R*_1_ = 0.0523 *w R*_2_ = 0.1382
*R* indices (all data)	*R*_1_ = 0.0993 *w R*_2_ = 0.1535
Largest diff. peaks [e Å ^−3^]	0.117, −0.947

**Table 3:** Selected bond lengths (d, Å).

Zr1 O1	1.960(4)	Zr2 O1	1.955(4)
Zr1 P1	2.652(2)	Zr2 P6	2.662(2)
P1 P2	2.210(2)	P6 P9	2.205(3)
P1 P4	2.203(3)	P6 P7	2.208(3)
P2 P3	2.238(3)	P9 P8	2.247(3)
P4 P3	2.245(3)	P7 P8	2.236(3)
P5 P3	2.222(3)	P8 P10	2.228(3)
P2 N1	1.701(9)	P9 N6	1.717(5)
P4 N2	1.697(5)	P7 N5	1.706(7)
P5 N4	1.698(7)	P10 N8	1.695(7)
P5 N3	1.703(6)	P10 N7	1.698(6)

**Table 4:** Selected bond angles (ω, deg).

Zr1 O1 Zr2	179.3(3)	P4 P3 P2	88.2(1)	P6 P7 P8	85.5(1)
O1 Zr1 P1	103.0(1)	Zr1 P1 P2	110.15(9)	P8 P9 P6	85.3(1)
P2 P1 P4	90.0(1)	Zr1 P1 P4	107.52(9)	P9 P8 P7	87.9(1)
P1 P4 P3	85.07(9)	O1 Zr2 P6	103.1(1)	Zr2 P6 P7	109.03(9)
P3 P2 P1	85.1(1)	P9 P6 P7	89.7(1)	P9 P6 Zr2	108.58(9)

**Table 5:** Most relevant X-ray and *ω*B97X-D/Def2TZV geometrical data of the main motif of complex **1** and reduced model **1′**.

Structure	1		1′
Method	X-ray	DFT	DFT
d(Zr1-Cp1)	2.500	2.527	2.527
d(Zr1-Cp2)	2.499	2.512	2.525
d(Zr2-Cp3)	2.509	2.511	2.525
d(Zr2-Cp4)	2.471	2.530	2.527
d(Zr1-O)	1.960	1.968	1.969
d(Zr2-O)	1.955	1.969	1.969
d(Zr1-P)	2.652	2.675	2.681
d(Zr2-P)	2.662	2.670	2.681
a(Zr1-O-Zr2)	179.3	177.5	180.0
a(O-Zr1-P)	103.0	103.6	103.7
a(O-Zr2-P)	103.1	104.1	103.7
a(O-Zr1-Cp1)	113.2	114.3	114.1
a(O-Zr1-Cp2)	109.0	109.8	113.5
a(O-Zr2-Cp3)	112.5	110.9	113.4
a(O-Zr2-Cp4)	106.6	87.7	114.2

**Table 6:** Average populations, $\bar{N}$, in average number of electrons, $\bar{e}$, associated with the ELF basins of **1′**. Subscript “t” refers to the total population of equivalent basins.

ELF Basins	$\bar{N}$	ELF Basins	$\bar{N}$
C(O)	2.11	C(P)	10.08
V_t_(O)	7.48	C(N)	2.11
C(Zr)	37.34	V(P)	2.24
V(Zr,P)	1.95	V(N)	1.78
C(C)	2.09	V(P,P)	1.78
V_t_(C)	1.21	V(N,P)	1.94
V(C,C)	2.64		

**Table 7:** Topological indicators and features that characterize the atomic interactions.

	*ρ*_cp_	∇ ^2^ *ρ*_cp_	G_cp_/*ρ*_cp_	H_cp_
Open-shell (covalent bonds)	Large	<0	<1	<0
Intermediate	Large	Arbitrary	≥1	<0
Polar covalent bond	Large	<0	>1	<0
Dative bond	Small	≥0	~1	≤0
Metallic bond	Small	~0	≤1	≤0
Closed-shell (ionic bonds)	Small	>0	≥1	>0

**Table 8:** QTAIM topological indicators at the BCP associated with the Zr-P, Zr-O and Zr-Cp regions at **1′**. *ρ*_cp_ is given in e·Å^−3^, ∇ ^2^*ρ*_cp_ in e·Å^−5^, and H_cp_, G_cp_ and V_cp_ in hartree·Å^−3^.

	*ρ*_cp_	∇ ^2^ *ρ*_cp_	G_cp_/*ρ*_cp_	H_cp_	H _cp_/*ρ*_cp_	|V_cp_|/G_cp_	DI
BCP1 (Zr-P)	0.058	0.044	0.453	−0.015	−0.262	1.578	0.564
BCP2 (Zr-O)	0.119	0.524	1.311	−0.026	−0.219	1.167	0.731
BCP3 ^a^ (Zr-Cp)	0.042	0.121	0.840	−0.004	−0.096	0.952	1.066 ^b^

^a^ Averaged properties at the 16 equivalent BCP3 associated with the Zr–C regions. ^b^ Defined as ∑[DI(Zr-C) + DI(Zr-H(C))].

**Table 9:** Percentage contributions to the integrated SF in complex **1′** at BCP1, BCP2 and BCP3 as reference points.

	BCP1 (Zr-P)		BCP2 (Zr-O)		BCP3 (Zr-Cp)	
	SF	%	SF	%	SF	%
Zr1	0.165	66.5	0.244	66.0	0.158	67.4
Zr2	0.044	17.7	0.067	18.3	0.043	18.2
O	−0.002	-	0.041	11.0	−0.001	-
P	0.023	9.4	0.001	0.3	0.001	0.3
Cp1	0.006	2.2	0.006	1.6	0.027	11.4
Cp2	0.005	2.1	0.006	1.5	0.005	2.1
Total	0.248	97.9	0.370	98.7	0.235	99.3

## Data Availability

Data are contained within the article or Supplementary Materials.

## Supplementary Materials

The following are available online. Physical characteristics of complex 1 (elemental analysis and IR spectrum). Table S1: Crystal X-ray diffraction data for complex 1.

## Sample Availability

Not available.
