# Supplementary material for: A Comprehensive Experimental and Theoretical Study on the [{(η5-C5H5)2Zr[P(µ-PNEt2)2P(NEt2)2P]}]2O Crystalline System
Source: Molecules. 2021 Nov 30;26(23):7282. doi: 10.3390/molecules26237282 (PMC8658821; doi:10.3390/molecules26237282)

SUPPLEMENTARY MATERIALS

A comprehensive experimental and theoretical study on the $[\{(\eta^5\text{-}C_5H_5)_2Zr[P(\mu\text{-}PNEt_2)_2P(NEt_2)_2P]\}_2O$ crystalline system

Agnieszka Łapczuk-Krygier*[1], Katarzyna Kazimierczuk[2], Jerzy Pikies[2], Mar Ríos-Gutiérrez*[3]

[1] Institute of Organic Chemistry and Technology, Cracow University of Technology, Warszawska St. 24, 31-155 Cracow, Poland, e-mail: a.lapczuk@.pk.edu.pl
[2] Chemical Faculty, Department of Inorganic Chemistry, Gdansk University of Technology, G. Narutowicza St. 11/12, Pl-80-233 Gdansk, Poland
[3] Department of Organic Chemistry, University of Valencia, Dr. Moliner 50, 46100 Burjassot, Valencia, Spain. E-mail: rios@utopia.uv.es
* Correspondence: a.lapczuk@.pk.edu.pl, rios@utopia.uv.es

SYNTHESIS

A standard Schlenk technique and an inert-atmosphere (argon) were employed for the synthesis and subsequent manipulations. Toluene and THF were dried over Na/benzophenone and distilled under nitrogen. A solution of $(Et_2N)_2PPSiMe_3Li$ [37](1mmol) in THF was added to $CpCp^*ZrCl_2$ [38](0,5mmol) in THF. The mixture turned initially dark red and rapidly discolored. Solvent was evaporated under vacuum, dry residue was dissolved in pentane and LiCl was filtered. This pentane solution was concentrated and after several days at -70°C small amount of red crystals of 1 deposited. When reaction mixture was heated (50°C, 4h) the yield of isolated **1** increased. Yield: 5-10% The attempts to study **1** with NMR in solution did not succeed because of low solubility of this compound.

X-RAY CRYSTALLOGRAPHY

Experimental diffraction data were collected on a KM4CCD kappa-geometry diffractometer, equipped with a Sapphire2 CCD detector. An enhanced X-ray MoKα radiation source with a graphite monochromator was used. Determination of the unit cells and data collection were carried out at 298 K. Data reduction, absorption correction, space group determination, solution and refinement were made using the CRYSALISPRO software package[39]. The structures were solved by direct methods and refined by full-matrix least-squares on F^2 (all data) using the SHELXL program package[40].

Crystallographic data for the structure reported here have been deposited with the Cambridge Crystallographic Data Centre (Deposition No. CCDC-1059036). This data can be obtained free of charge via http://www.ccdc.cam.ac.uk/perl/catreq.cgi (or from the CCDC, 12 Union Road, Cambridge CB2 1EZ, UK; Fax: (+44) 1223-336-033; e-mail: deposit@ccdc.cam. ac.uk.

PHYSICAL CHARACTERISTICS OF COMPLEX 1

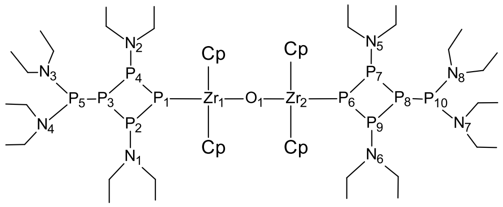

$C_{52}H_{100}N_8OP_{10}Zr_2$, red crystals obtained in 5-10% yield; Elemental analysis of (1): Anal. Calc. for $C_{52}H_{100}N_8OP_{10}Zr_2$: C, 46,42; H, 7,49, N 8,33. Found: C 47,72; H 7,78; N 8,58%

IR

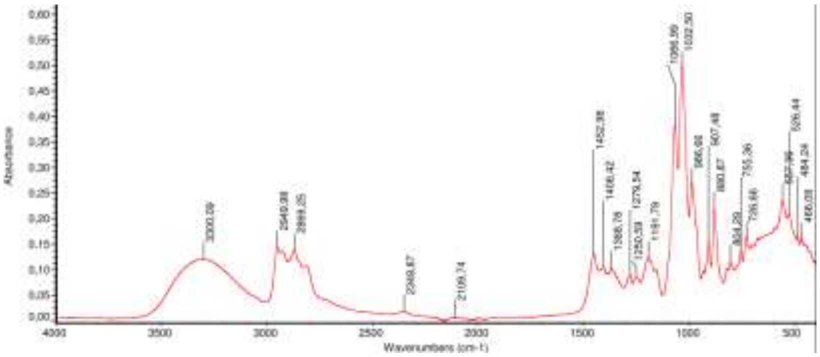

CRYSTAL X-RAY DIFFRACTION

Table S1. Crystal X-Ray diffraction data for complex 1

Empirical formula	$C_{52}H_{100}N_8OP_{10}Zr_2$
Formula weight	1345,54
Temperature /K	120(2)
Wavelength /Å	0.71073 (Mo Kα)
Crystal system	monoclinic
Space group	$P\ 2_1/n$
a /Å	196452(14)
b /Å	17.8701(12)
c /Å	20.7963(14)
α/deg	90
β /deg	112.953(7)
γ /deg	90

V /Å³	6722.7(8)
Z	4
D_c/Mg m⁻³	1.329
μ /mm⁻¹	0.588
F(000)	2824
Crystalsize /mm	0.0673 x 0.0423 x 0.0141
*Θ*range/deg	2.25 to 25.5
Index ranges	-21<=h<=23<br>-21<=k<=21<br>-25<=l<=23
Reflections collected / unique	47963 / 12510[*R*(int) = 0.1454]
Data / restraints / parameters	12510 / 0 / 658
Goodness of fit on F^2	0.805
Final *R* indices [*I*>2σ(*I*)]	R_1 = 0.0523<br>$w R_2$ = 0.1382
R indices (all data)	R_1 = 0.0993<br>$w R_2$ = 0.1535
Largest diff. peaks[e Å⁻³]	0.117, -0.947

STRUCTURE OF ***1***

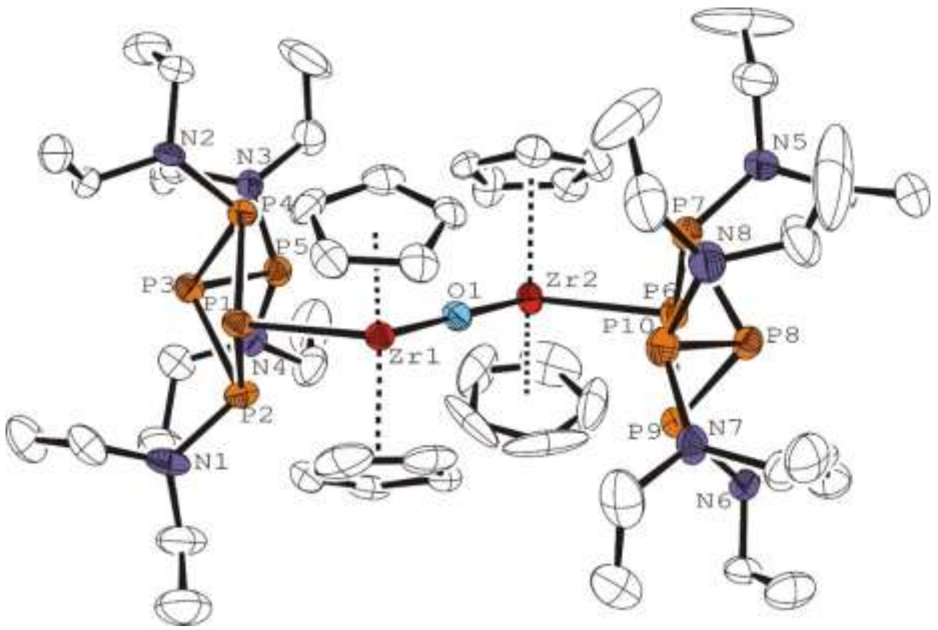

Supplement: Supplementary file 1 [file molecules-26-07282-s001.zip › molecules-1427695-supplementary.pdf]
